# Development of an easy-to-use urease kit for detecting *Helicobacter pylori* in canine gastric mucosa

**DOI:** 10.14202/vetworld.2021.1977-1987

**Published:** 2021-07-30

**Authors:** Chularat Hlaoperm, Kiattawee Choowongkomon, Chantima Pruksakorn, Jatuporn Rattanasrisomporn

**Affiliations:** 1 Graduate Program in Animal Health and Biomedical Sciences, Faculty of Veterinary Medicine, Kasetsart University, Bangkok 10900, Thailand; 2 Department of Companion Animal Clinical Sciences, Faculty of Veterinary Medicine, Kasetsart University, Kamphaeng Saen Campus, Nakhon Pathom 73140, Thailand; 3 Department of Biochemistry, Faculty of Science, Kasetsart University, Bangkok 10900, Thailand; 4 Department of Veterinary Microbiology and Immunology, Faculty of Veterinary Medicine, Kasetsart University, Bangkok 10900, Thailand

**Keywords:** dog, *Helicobacter pylori*, rapid urease test

## Abstract

**Background and Aim:**

*Helicobacter pylori* is an important pathogen in humans and animals involved in chronic gastritis, leading to the development of gastric cancer. Urease produced by *H. pylori* is an enzyme that promotes bacterial colonization and can be used clinically as a biomarker of *H. pylori* infection as part of a rapid urease test (RUT). A test with high specificity (95-100%) would be more convenient and faster than histopathology, bacterial culture, and polymerase chain reaction (PCR). The aim of this study was to develop a simple, cheap, and fast kit for detecting *H. pylori* infection in the gastric mucosa of canines, which can be used in clinical practice for diagnosing infection with this bacterium.

**Materials and Methods:**

The RUT assays developed were prepared using 1% agar, 1% sodium phosphate monobasic, and 1% urea followed by the addition of 3% methyl red indicator. The cutoff value of sensitivity of the RUT assay was established using the urease of *H. pylori* ATCC 43504 and color change was monitored for 24 h. Comparisons of the sensitivity to *H. pylori* ATCC 43504 were made between the developed RUT assays and the Hp Fast™ commercial kit. Then, the limit of detection for *H. pylori* ATCC 43504 number was analyzed by the SYBR Green real-time PCR assay to measure the copy number of the *ure*C gene. Gastric biopsy samples from the antrum, body, and fundus of the stomach were collected from eight canines presenting with vomiting and gastroenteritis. Analyses were performed on fresh samples using the developed RUT assays and the Hp Fast™ commercial kit, which were read within 24 h; then, the results were confirmed with SYBR Green real-time PCR. The specificity of the RUT assays was tested with a number of different bacteria, including *Staphylococcus pseudintermedius*, *Proteus* spp., *Pseudomonas aeruginosa*, *Klebsiella pneumoniae*, *Enterococcus* spp., *Escherichia coli*, and *Salmonella* spp.; *H. pylori* ATCC 43504 was used as a positive control.

**Results:**

The results showed that the developed assays were sensitive to the urease enzyme at 0.1 mg/mL. The lowest detection limit of this assay for *H. pylori* ATCC 43504 was found to be 10^2^ copies at 30 min. The sensitivity of detection of *H. pylori* in gastric biopsies of canines occurred in a minimum of 30 min. The RUT showed similar results to the Hp Fast™ commercial kit. In the developed RUT, the color change of the test from red to yellow could be clearly distinguished between the color of the positive test and the negative one; however, in the commercial Hp Fast™, it was difficult to observe the gel color changein the negative pH range of 5.8 and the positive pH of 6.5. The developed RUT was specific for *H. pylori* and did not detect any of the other tested bacteria. The test kit can also be stored for 6 months at 4°C.

**Conclusion:**

The sensitivity of the developed assays allowed the detection of urease enzyme at a minimum concentration of 0.1 mg/mL. Our RUT could also detect *H. pylori* from one in eight canine specimens at a minimum of 10^2^ copies within 30 min. This RUT is specific to *H. pylori* as it did not detect any of the other tested bacteria.

## Introduction

*Helicobacter* spp. is a spiral-shaped, Gram-negative pathogenic bacterium. Its primary habitat is the gastric mucosa of not only humans but also animals [1]. *Helicobacter* species have been reported in both wild and domestic mammals having various dietary habits, such as dogs, cats, mice, swine, cattle, and sheep [2]. The various species of *Helicobacter* and different strains within the same species may not necessarily cause similar changes in gastric regions. *Helicobacter* *pylori* causes changes in the mucosa of the human stomach because of its mobility and its ability to resist high acidity [3]. *H. pylori* has been identified as a serious cause of peptic ulcerative diseases (gastric and duodenal ulcers), gastritis, chronic and gastric cancer, and even gastric lymphoma [4]. In 2005, Barry Marshall and Robin Warren were awarded the Nobel Prize in Physiology for their work on *H. pylori*. According to the Nobel Committee, this prize was given for their discovery of the role played by *H. pylori* in gastritis and peptic ulcer[5]. This is extraordinary on the grounds that *H. pylori* is the only bacterium known to cause gastric cancer [6]. Therefore, this bacterium has become the target of a large number of studies on the human stomach.

In 1994, the World Health Organization and the International Agency for Research on Cancer designated *H. pylori* as a Class 1 cancer-causing agent and a definite cause of gastric adenocarcinoma in humans [7]. *H. pylori* is estimated to affect 50% of the global population, having a presence in both developed and developing nations; its prevalence can be higher than 70% in developing nations [8]. In Thailand, the incidence of *H. pylori* infection in humans was reported to be 57% [9]. *H. pylori* disease occurs essentially within families; it has been asserted that individual-to-individual spread is the most probable method of transmission. Fecal–oral, oral–oral, and gastro–oral transmission routes are likely, especially since *H. pylori* can be discharged through defecation, spit, and vomitus. Given the role of *H. pylori* contamination in gastrointestinal infection, an exact diagnosis of *H. pylori* disease is a necessary foundation for treating different gastrointestinal indications and forestalling genuine complications [10].

Against this background, the diagnostic method for detecting infection in patients with gastritis is important. Standard culture, which is a method for isolating *H. pylori* from stomach biopsies of patients, may take up to 5 to 7 days to achieve optimal growth, and the cultivation of *H. pylori* is labor-intensive and expensive [11]. Then, the growth of *H. pylori* is confirmed according to biochemical tests (positive for oxidase, catalase, and urease) [12]. To date, several more effective assays of *H. pylori* have been developed. Specifically, there are two groups of diagnostic tests for *H. pylori*, namely, invasive tests based on endoscopy in patients, for example, the rapid urease test (RUT), bacterial culture, and histopathology, and non-invasive tests, such as the urease breath test, serology, and the stool antigen test, which are specific and sensitive for *H. pylori* but quite expensive [10,13].

*H. pylori* produces urease, an enzyme that promotes bacterial colonization, and is used clinically as a biomarker for *H. pylori* infection as part of the rapid RUT, having specificity of 95-100% for infectious agents, depending on the severity of infection. This method is more convenient and faster than histopathology, bacterial culture, and polymerase chain reaction (PCR) [5,10]. In addition to *H. pylori*, other *Helicobacter* species may infect humans and be associated with gastritis in animals such as canines and have been identified as non-*H. pylori Helicobacter* [14]. Infection with *Helicobacter* spp. was reported at a high prevalence in the gastric mucosa of canines; *Helicobacter* spp. DNA was detectable in the oral cavity of over 70% of canines. These findings support the possibility of oral–oral transmission between canine oral cavities, which may act as a source of non-*H. pylori* *Helicobacter* spp. infection in humans [14]. Against this background, we were interested in developing a test kit for *H. pylori* in canines with the RUT because it is more convenient and rapid than the other methods mentioned above.

The aim of this study was thus to develop accurate RUT assays to diagnose *H. pylori* infection in the gastric mucosa of canines using a pH-sensitive indicator. If present, *H. pylori* urease metabolizes urea, leading to increased pH, as a result, the test kit developed was positive. The developed RUT assays are cheaper than commercial kits, can be used in clinical practice to detect *H. pylori* in canine gastric mucosa, and can be applied in the clinical diagnosis of *H. pylori* in human gastric mucosa.

## Materials and Methods

### Ethical approval

The animal use protocol was reviewed and approved by the Kasetsart University Animal Use Committee for the Use of Animals in Research and Education, under the protocol number ACKU62-VET-062.

### Study period and location

The study was conducted from February 2019 to November 2020 at the Faculty of Veterinary Medicine, Veterinary Teaching Hospital and Faculty of Science, Kasetsart University.

### Animals and samples collection

Eight canines (four males and four females) which underwent gastroscopy were used in this study. The gastric biopsy samples from the antrum, body, and fundus parts of the stomach, were collected from canines presenting with vomiting and gastroenteritis. The evaluation of the gastric mucosa was based on the procedures of the World Small Animal Veterinary Association and the previous studies of Sousa *et al*. [15]. Animals’ history information was gathered including age, sex, breed, as well as the results of diagnostic records and final diagnosis. Three specimens were taken from each dog as samples to test with the developed RUT assays.

### Preparation of the developed RUT assays

The developed RUT assays were prepared using 1% agar (Thermo Scientific™, USA), 1% sodium phosphate monobasic (Sigma-Aldrich, USA), and 1% urea (KemAus, Australia). These components were dissolved in distilled water, boiled to completely dissolve the materials, and autoclaved at 121°C and 15 psi for 15 min. The agar was cooled to 50-55°C and then 3% methyl red indicator was added, after which the agar solution was mixed thoroughly. One milliliter was distributed per sterile plastic container (containers for contact lenses). The containers were left on a sterile surface until the agar had solidified. The lids of the containers were then closed and stored in a refrigerator at 4-8°C until used to develop the RUT assays.

### Determination of the optimal cutoff value for sensitivity to urease of the developed RUT

The test to determine the lowest urease concentration detectable (producing a color change) in the RUT employed the urease in *Canavalia ensiformis* (Jack bean) powder (Sigma-Aldrich). The urease was diluted in distilled water at concentrations of 0.1, 0.01, 0.001, 0.0001, 0.00001, and 0.000001 mg/mL; approximately 60 μL of each concentration was used in the developed RUT assays. These assays were observed for a color change at 1, 3, 5, 10, 20, and 30 min and 1, 2, 3, 4, 5, 6, and 24 h. The negative control was 60 μL of Deionized (DI) water, which substituted for the urease.

### Bacterial strains and culture conditions

Reference strains including *H. pylori* ATCC 43504 were used to validate the developed RUT assays. *H. pylori* ATCC 43504 was cultured on tryptic soy agar (Difco; BBL, USA) with 5% defibrinated sheep blood (Thermo Scientific™, Thailand). Agar slants were incubated at 37°C for 3 days under a microaerobic atmosphere using a gas pack system (Thermo Scientific™, Thailand). Other microorganisms used for the specificity test included *Staphylococcus pseudintermedius*, *Proteus* spp., *Pseudomonas aeruginosa*, *Klebsiella pneumoniae*, *Enterococcus* spp., *Escherichia coli*, and *Salmonella* spp., which were cultured in brain–heart infusion broth (Difco, USA) at 37°C for 24 h.

### Optimal cutoff value for sensitivity of a RUT

The test determined the lowest *H. pylori* number that would make the developed RUT assays change color, using *H. pylori* ATCC 43504 in trypticase soy broth (Difco; BBL, USA) medium passed through ten-fold serial dilution in phosphate-buffered saline (PBS) buffer. Approximately 60 μL of the solution was dispensed into each of the developed RUT assays and the Hp Fast™ commercial kit. The lids of the containers were closed, samples were incubated at room temperature (25°C), and color change was observed at 1, 3, 5, 10, 20, and 30 min and every hour up to 24 h. The results were compared between the developed RUT assays and the Hp Fast™ commercial kit. The negative control was *H. pylori* ATCC 43504 diluted 10 times in 1X PBS buffer and heated at 100°C for 30 min. The genomic DNA of *H*. *pylori* ATCC 43504 in solution was extracted with the GF-1 Bacterial DNA Extraction Kit (Vivantis, Malaysia), in accordance with the manufacturer’s instructions. The limit of detection was analyzed by the SYBR Green real-time PCR assay to measure the copy number of the *ure*C gene to detect *H. pylori*.

Next, 3.0 mm biopsy samples of fresh gastric mucosa samples from the stomach antrum, body, and fundus of each canine were collected and individually tested in the developed RUT assays and by the Hp Fast™ commercial kit. Samples in the developed RUT assay that changed color from red to yellow were considered positive for *H. pylori*. The reading of results was performed at 1, 3, 5, 10, 20, and 30 min and 1, 2, 3, 4, 5, 6, up to 24 h, and the timing of color changes was analyzed for a possible association with the amount of bacteria in the sample. Genomic DNA from stool specimens of the canines was extracted using the E.Z.N.A.^®^ Tissue DNA Kit (Omega BIO-TEK, USA), in accordance with the manufacturer’s instructions. Genomic DNA was detected by SYBR Green real-time PCR in all samples to confirm the results and then considered together with the histopathological diagnosis.

### DNA template preparation for standard curve

*H. pylori* ATCC 43504 was analyzed with the GF-1 Plasmid DNA Extraction Kit (Vivantis, Malaysia), in accordance with the manufacturer’s instructions. PCR amplification of the *ureC* gene of *H. pylori* ATCC 43504 was performed using the specific forward primer 5’-TTATCGGTAAAGACACCAGAAA-3’ and reverse primer 5’-ATCACAGCGCATGTCTTC-3’. In this study, we used primers specific for the *ure*C gene of *H. pylori* ATCC 43504 [16]. Total DNA of *H. pylori* ATCC 43504 at 100 ng/μL (1 μL) was added to a final volume of 10 μL. The reaction mixture contained 5.75 μL of DI water, 10×0.3mM μL (1 μL) PCR buffer, 2.5 μM μL (1 μL) dNTP, 0.25 μL of Taq DNA Polymerase, 5 μM (0.5 μL) forward and reverse primers and the reaction mixture. PCR amplification consisted of initial denaturation at 95°C for the target DNA for 3 s, following by 30 cycles of denaturation at 95°C for 30 s, annealing at 55°C for 30 s, and extension at 72°C for 15 s. The final extension was performed at 72°C for 5 min to ensure the full extension of the PCR product. To check for amplification contaminants, the assay was run with no template DNA; 1 μL of DI water was substituted for the template. A 146 bp product resulted from the reaction.

The PCR product of 146 bp was cloned into p-GEMT-Vector (Promega Corporation, USA) following the manufacturer’s instructions and transformed into host strain *E. coli* BL21 competent cells. Automated DNA sequencing further confirmed that the expected *ure*C gene fragment had been cloned into the p-GEMT-Vector, in accordance with the website of the NCBI (National Center for Biotechnology Information) using the BLAST^®^ program.

The plasmid DNA from cloning was extracted using the GF-1 Plasmid DNA Extraction Kit (Vivantis, Malaysia) following the manufacturer’s instructions. The DNA solution’s absorbance was measured at 260 nm 3 times with a NanoDrop™ Spectrophotometer (Thermo Scientific™, USA). Next, 100 ng/mL circular plasmid from cloning was analyzed for the copy number of the *ure*C gene following formula 1 using the DNA Copy Number and Dilution Calculator program (Thermo Scientific™).


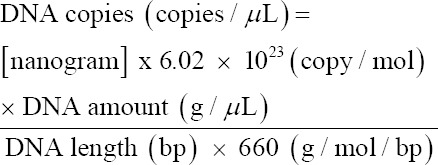


DNA amount (g/μL)=The measured plasmid concentration value

DNA length (bp)=Plasmid length and the connected genes

The measured plasmid copy numbers of the *ure*C gene was diluted in a ten-fold series in DI water at concentrations of 10^1^-10^7^ copy numbers for a standard curve.

### SYBR Green real-time PCR for calculating the *ureC* gene copy number in *H. pylori* ATCC 43504

For the real-time PCR reaction, the Maxima SYBR Green/ROX qPCR Master Mix (Bio-Rad, USA) was used to determine the copy number of the *H. pylori* *ure*C gene. The genomic DNA was extracted from the *H. pylori* ATCC 43504 strain using the GF-1 Bacterial DNA Extraction Kit (Vivantis, Malaysia) following the manufacturer’s instructions. It was diluted 10 times in concentrated 1X PBS buffer. The lowest concentrations of *H. pylori* ATCC 43504 that resulted in a positive color change in the developed test kit were calculated by SYBR Green real-time PCR compared with plasmid DNA extracted from cloning, which was used as a standard solution at gene copy numbers of 10^7^, 10^6^, 10^5^, 10^4^, 10^3^, 10^2^, and 10^1^. SYBR Green real-time PCR amplification of the *ure*C gene of *H. pylori* ATCC 43504 was performed using the specific forward and reverse primers mentioned above. The reaction mixture contained 7.5 mL of DI water, 5 mM (1.5 mL) forward and reverse primers, and 12.5 mL of (2´) Maxima SYBR Green/ROX qPCR Master Mix (Bio-Rad, USA). Two microliters of DNA plasmid and genomic DNA of *H. pylori* ATCC 43504 were added to each concentration to a final volume of 25 mL per reaction. The amplification consisted of initial denaturation at 50°C for 5 min, followed by 30 cycles of denaturation at 94°C for 30 s, annealing at 59°C for 30 s, and extension at 72°C for 30 s. This was, in turn, followed by melting curve analysis by setting the initial temperature to 65°C increasing by 0.5°C every 5 s to ensure the full extension of the PCR product. A 146 bp product resulted from the reaction. The negative controls were 2 mL of DI water, which substituted for the template.

### Detection specificity of the developed RUT assays

The microorganisms were diluted with PBS buffer at 150×10^6^ bacterial cells/mL. McFarland Standard 0.5 was used for the test of the specificity of the developed RUT assays. A number of microorganisms can produce urease, including *S. pseudintermedius*, *Proteus* spp., *P. aeruginosa*, and *K. pneumoniae*. In this study, the urease-negative group included *Enterococcus* spp., *E. coli*, and *Salmonella* spp., while *H. pylori* ATCC 43504 was used as a positive control. Approximately 60 µL of the bacterial solutions were dispensed in the developed RUT assays to detect specificity of the developed RUT assays in other bacteria that are not *H. pylori*. The developed RUT assays were observed for color change at 1, 3, 5, 10, 20, and 30 min and at 1, 2, 3, 4, 5, and 6 h, until 24 h.

### Stability test of the developed RUT assays

To test the shelf life of the developed assays, four groups were established. Group 1 was supplemented with the preservative 1% methyl paraben (Salicylates And Chemicals Private Limited, India) followed by storage at 25°C. Group 2 was stored at 25°C without the preservative. Group 3 was treated with the preservative and stored at 4°C. Group 4 was stored at 4°C without the preservative. All four groups were tested for detection of the urease enzyme at a concentration of 0.1 mg/mL and volume of 60 mL, as revealed by color change. The test lasted 6 months.

## Results

### Establishment of RUT sensitivity for detection of urease

**Figure-1:**
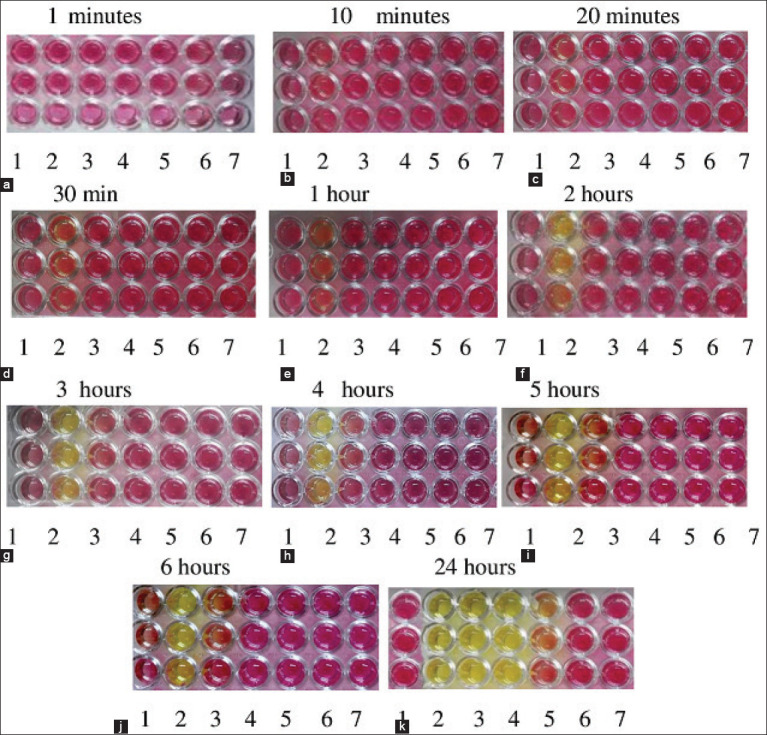
The color change occurring in the developed rapid urease test (RUT) assays to detect urease (assay was repeated 3 times), a, b, c, d, e, f, g, h, i, j, and k changed color from red to yellow at 1 min, 10 min, 20 min, 30 min, and 1 h, 2 h, 3 h, 4 h, 5 h and 6 h, through 24 h, respectively. Well number 1 contained deionized water (Negative control). Well numbers 2, 3, 4, 5, 6 and 7 were urease concentrations 0.1, 0.01, 0.001, 0.0001, 0.00001 and 0.000001 mg/mL, respectively.

Tests to determine the lowest urease concentration that would change the color in the developed assay were performed using urease from *C. ensiformis* powder (Sigma-Aldrich) diluted to concentrations of 0.1, 0.01, 0.001, 0.0001, 0.00001, and 0.000001 mg/mL. The developed test kit contains methyl red as a color indicator, which changes from red to yellow when the pH increases. The results showed that, at the enzyme concentration of 0.1 mg/mL, a noticeable color change occurred in the period of 10–30 min and this color remained clear from 2 h to 24 h. The enzyme concentrations of 0.01, 0.001, and 0.0001 mg/mL caused a color change on incubation for 24 h. At enzyme concentrations of 0.00001 and 0.000001 mg/mL and deionized water, no color change occurred (Figure-1 and Table-1).

**Table-1:** Results of the color change test to determine the sensitivity of the developed RUT to detect urease.

Urease enzyme (mg/mL)	Time to change color					

	<10 min	1 h	1-2 h	2-4 h	4-6 h	24 h
0.1	−	+	+	+	+	+
0.01	−	−	−	−	−	+
0.001	−	−	−	−	−	+
0.0001	−	−	−	−	−	+
0.00001	−	−	−	−	−	−
0.000001	−	−	−	−	−	−

The plus (+) indicates the concentration of urease that caused a color change from red to yellow and the minus sign (−) indicates a negative result. RUT=Rapid urease test

### Sensitivity of the developed RUT for *H. pylori* ATCC 43504

**Figure-2:**
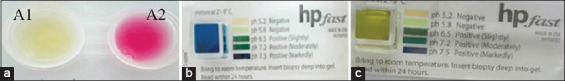
The smallest number of *Helicobacter pylori* 43504 to cause a color change in the developed rapid urease test (RUT) was compared with commercial kit HP Fast™ incubated at 25°C and read within 24 h. (a1) *H. pylori* ATCC 43504 from a stock culture was tested by the developed RUT. (a2, c) Negative control (b) *H. pylori* ATCC 43504 from a stock culture tested by the commercial kit HP Fast™.

After 24 h of testing with serially diluted 10-fold *H. pylori*, both the developed RUT and the Hp Fast™ commercial kit detected *H. pylori* ATCC 43504. The developed test changed color from red to yellow after 30 min. The commercial kit was also positive as indicated by a change from yellow to blue. The negative control was *H. pylori* ATCC 43504 diluted 10 times in 1X PBS buffer and heated at 100°C (Figure-2).

**Figure-3:**
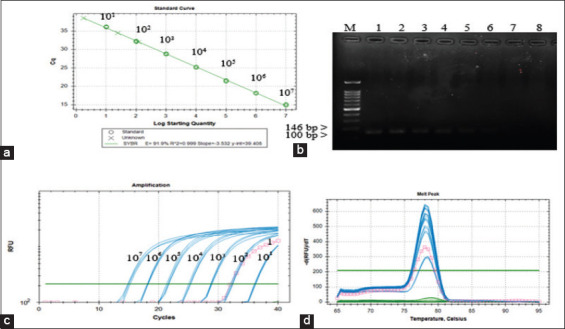
The analytical sensitivity (detection limit) of the developed rapid urease test (RUT). Detection values were calculated by SYBR green real time-PCR. (a) The *ure*C gene copy numbers for the standard curve were calculated with formula 1 with a range from 10^1^ to 10^7^ copy numbers (green line). (b) Gel electrophoresis specific to *ure*C gene 10^7^-10^1^ copies; this range still had a specific product of 146 bp, lane M, 100-bp markers; lanes 1-7, 10^7^-10^1^ copies of the *Helicobacter pylori* *ure*C gene, respectively; lane 8, deionized water. (c) The analytical sensitivity of the developed RUT, detection values calculated by SYBR green real time-PCR, detected 10^2^ copy numbers (pink line). (d) Mean of Tm at 78°C indicated specific amplification of *H. pylori* ATCC 43504.

The genomic DNA was extracted from each serially diluted 10-fold *H. pylori* ATCC 43504 and SYBR Green real-time PCR was used to determine the limit of detection of the developed RUT. SYBR Green real-time PCR assay used to measure the *ure*C gene copy number to detect *H. pylori* ATCC 43504 determined the presence of one copy of the *ure*C gene per bacterium. The *ure*C gene copy numbers for a standard curve were calculated using formula 1 with a range of copy numbers of 10^7^-10^1^; three replicates of each dilution were used. In addition, linear regression analysis showed R²=0.999 and efficiency (E) of 0.97%. Linear regression analysis, plotting the cycle number versus the log concentration of the amplicon, gave a straight-line plot and a correlation coefficient (Figure-3a). Gel electrophoresis of the *ure*C gene for 10^7^-10^1^ copies indicated that this range still had specific product amplification, and demonstrated that amplification of *ure*C occurred at all template concentrations tested (Figure-3b). The result was interpreted as positive based on a Cq value ≤35. This value tells how many cycles it took to detect a real signal from the samples. From SYBR Green real-time PCR, a reaction curve was established for each sample and thus many Cq values were obtained.

To determine the analytical sensitivity (detection limit) of the developed RUT, detection values were calculated for *H. pylori* detection number by SYBR Green real-time PCR. This showed that the lowest detected concentration on the dilution of genomic DNA of *H. pylori* ATCC 43504 was a copy number of 10^2^, as shown in Figure-3c. Mean melting temperature (Tm) at 78°C indicated specific amplification of *H. pylor*i ATCC 43504 (Figure-3d).

### Developed RUT assays with gastroscopic biopsy specimens

**Figure-4:**
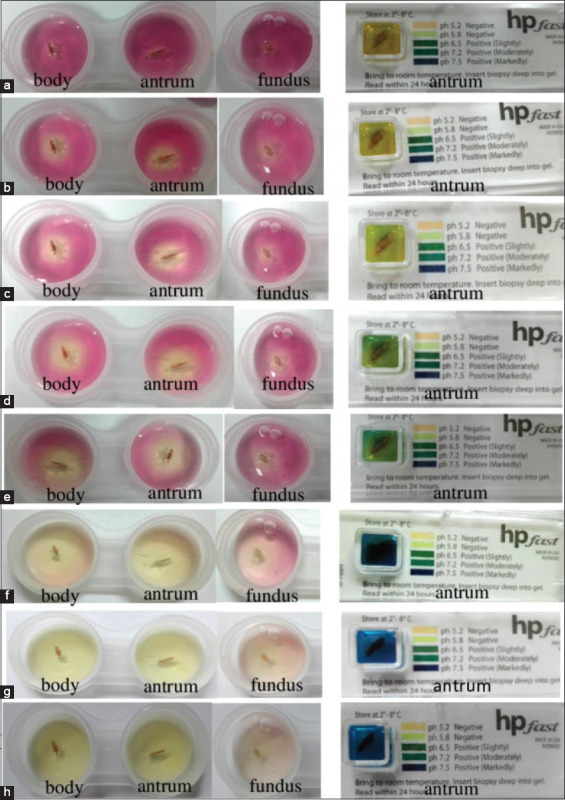
Gastric biopsy samples of the body, antrum and fundus were analyzed by the developed rapid urease test and compared with the HP Fast™ commercial kit. (a, b, c, d, e, f, g, and h) caused a color change from red to yellow at 3 min, 30 min, and 1 h, 2 h, 3 h, 4 h, and overnight, curing up to 24 h, respectively.

The gastric biopsy samples from eight canines were tested with the developed RUT assays and the Hp Fast™ commercial kit, which found that one of the canines gave a positive result. It was found that the developed test could detect *H. pylori* at 30 min (Figure-4b). The color change was more clearly noticeable over the period from 4 to 24 h, with positive results from the gastric biopsies of the stomach body, antrum, and fundus in both the developed RUT assays and the Hp Fast™ commercial kit (Figure-4f-h). The Hp Fast™ commercial kit gave positive results indicated by a color change noticeable at 2 h and pH 6.5; the color change became clearer over the period from 4 to 24 h. The same results were achieved with the developed RUT assays. *H. pylor*i infection can cause peptic ulcer disease usually localizing in the antrum, leading to increased pH, as a result, the test kits were positive [17]. The Hp Fast™ commercial kit was used for comparison with the developed RUT.

Table-2 shows the results of the developed RUT assays on biopsies; one of the canines gave a positive result. The results were confirmed by SYBR Green real-time PCR. The *ure*C gene copy number was used to determine the number of *H. pylori* in biopsy samples, which were positive at a copy number of 4.78×10^9^ and produced a Cq value of 11.64. When a dog with a history of chronic vomiting was tested histopathologically, it was found that it had symptoms of chronic enteritis duodenitis *H. pylori* infection alters acids in the duodenum, produces abnormal duodenal movement that cause chronic enteritis, and leads to manifestations of ulcerative colitis [17,18].

**Table-2:** The results of the developed RUT, Hp Fast™ and SYBR Green real time-PCR assays and histopathological diagnosis based on the biopsy samples from eight canines.

Canine	Developed RUT assays	Hp Fast™	Real time-PCR	Histopathological diagnosis
1	−ve	−ve	−ve	Normal gastric mucosa
2	−ve	−ve	−ve	Normal gastric mucosa
3	+ve	+ve	+ve	Chronic enteritis duodenitis
4	−ve	−ve	−ve	N/A
5	−ve	−ve	−ve	Normal gastric mucosa
6	−ve	−ve	−ve	N/A
7	−ve	−ve	−ve	No inflammatory, hyperplasia or neoplasia
8	−ve	−ve	−ve	Ulcerative eosinophilic gastritis

+ve=positive, −ve=negative and N/A=not available, RUT=Rapid urease test

### The specificity of the developed RUT assays

**Figure-5:**
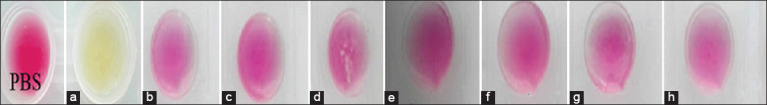
Results of the specificity test of the developed rapid urease test (RUT) with bacterial concentration at 150×10^6^ cells/mL (McFarland Standard 0.5). Testing was with *Helicobacter pylori* ATCC 43504 and the results of the RUT assays were positive indicated by the color change from red to yellow (a). Then, b, c, d, e, f, g, h were *Staphylococcus pseudintermedius*, *Proteus* spp., *Pseudomonas aeruginosa*, *Klebsiella pneumoniae*, *Enterococcus* spp., *Escherichia* *coli*, and *Salmonella* spp., respectively, and all tested negative.

Specificity tests of the developed RUT assays used a bacterial concentration of 50×10^6^ cells/mL (McFarland Standard 0.5) at a volume of 60 mL (Table-3). The negative control was 1X PBS buffer. Samples were incubated for 24 h. The results were negative for *S. pseudintermedius*, *Proteus* spp., *P. aeruginosa*, *K. pneumoniae*, *Enterococcus* spp., *E. coli*, and *Salmonella* spp (Figure-5b-h). *H. pylori* ATCC 43504 (positive control) was positive, with the developed test color changing from red to yellow after 50 min (Figure-5a).

**Table-3:** Results of specificity tests of the developed RUT with a bacterial concentration of 150×10^6^ cells/mL (McFarland Standard 0.5).

Bacterial species	Color change of the developed test												

	min						h						
	
	5	10	20	30	40	50	1	2	3	4	5	6	24
*H. pylori* ATCC 43504	−	−	−	−	−	+	+	+	+	+	+	+	+
*S. pseudintermedius*	−	−	−	−	−	−	−	−	−	−	−	−	−
*Proteus* spp.	−	−	−	−	−	−	−	−	−	−	−	−	−
*P. aeruginosa*	−	−	−	−	−	−	−	−	−	−	−	−	−
*K. pneumoniae*	−	−	−	−	−	−	−	−	−	−	−	−	−
*Enterococcus* spp.	−	−	−	−	−	−	−	−	−	−	−	−	−
*E. coli*	−	−	−	−	−	−	−	−	−	−	−	−	−
*Salmonella* spp.	−	−	−	−	−	−	−	−	−	−	−	−	−

+=Positive, −=Negative, *H. pylori=Helicobacter pylori,*

*S. pseudintermedius=Staphylococcus pseudintermedius, P. aeruginosa=Pseudomonas aeruginosa,*

*K. pneumonia=Klebsiella pneumoniae, E. coli=Escherichia coli*, RUT=Rapid urease test

### Stability test of the developed RUT

**Figure-6:**
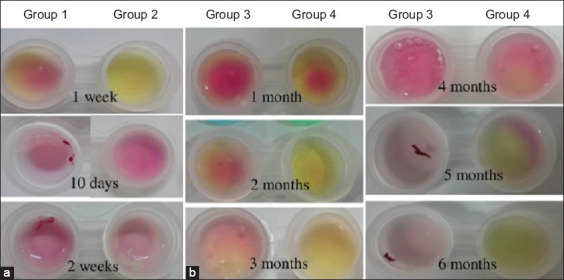
Results of stability test of the developed rapid urease test at 6 months. (a) Gels were stored at 25°C for 2 weeks; both groups of gels were dry. (b) Gels were stored at 4°C, Group 4 was able to detect urease for up to 6 months.

A test of the stability of the developed RUT was conducted at 6 months with urease at a concentration of 0.1 mg/mL and a volume of 60 mL. Then, color change was observed and the results were recorded. In Group 1, 1% methyl paraben (Salicylates And Chemicals Private Limited) was added at 25°C. Group 2 was tested without the methyl paraben and kept at 25°C. The results showed that Group 1 exhibited a slower color change than Group 2; when kept for 2 weeks, the gels of both groups were dry because the high temperature caused evaporation, and the tests could not be continued (Figure-6a). In Group 3, the developed RUT was supplemented with 1% methyl paraben and stored at 4°C. In Group 4, storage was performed at 4°C without methyl paraben. In Group 3, the color change occurred after 3 months in storage, but was slower than in Group 4. In Group 4, urease could be detected for 6 months (Figure-6b).

## Discussion

*H. pylori* exhibits the unusual characteristic of producing large amounts of urease [19]. Thus, we developed the RUT assays carried out in a small amount of agar containing urea and a pH indicator. If urease is present, the urea is broken down into ammonia and carbamate, thus raising the pH, and the pH indicator changes from red to yellow. The reaction usually occurs within 24 h. The developed RUT could detect a minimum of 0.1 mg/mL urease after 30 min of incubation, and the change of color from red to yellow was clearly observable within 2 h (Figure-1d-f).

Next, we investigated the sensitivity (detection limit) of the developed RUT for *H. pylori* ATCC 43504 and compared the results with those from the commercial kit Hp Fast™, read within 24 h. The developed test produced a color change from red to yellow at 30 min up to 24 h, and this correlated with the positivity in the commercial kit Hp Fast™, in which the color changed from yellow to blue (Figure-2). The lowest concentration of *H. pylori* ATCC 43504 was selected to calculate the *ure*C copy number by SYBR Green real-time PCR, in view of the fact that there is just one copy of *ure*C per bacterium, so it is suitable for calculating the load of *H. pylori* [20]. The quantitative real-time PCR assay for *ure*C gene was used to calculate the *H. pylori* number to determine the limit of detection of the developed RUT. The PCR product obtained from the real-time PCR reaction of each bacterial species has an appropriate melting temperature (Tm) that was displayed with DNA peak in the graph, which determines the conditions under which these will bind to target gene [21]. The results of the melting point curve show that there was no mispriming in the test; the use of Tm at 78°C ensures that the primer used only amplifies the *ure*C target gene, as indicated by the formation of a single peak (Figure-3d). The mean Cq was calculated for each bacterial concentration, the Cq values ≤35 were considered as positive results. The presence of a DNA band specific for the *ure*C gene by gel electrophoresis was indicated by a product size of 146 bp (Figure-3b). In this study, we used primers specific to the *ure*C gene of *H. pylori* ATCC 43504, as in a previous study [16]. The results of analysis of the nucleotide sequence database through the NCBI website found that the primers were specific to the *ure*C gene of *H. pylori* ATCC 43504 at 99.29%.

SYBR Green real-time PCR analyses revealed that the developed RUT can detect infection at a copy number of 10^2^; the Cq value was 32 and the Tm 78°C (Figure-3c). A copy number lower than 10^2^ will give a negative result, based on one copy of the *ure*C gene per bacterium [20]. This result is in accordance with a previous study [22], in which the results of a RUT for concentrated urease production of *Helicobacter* species were found to be positive at 10^2^ copies. In the developed RUT, the color change of the test from red to yellow could clearly distinguish positivity and negativity, but in the commercial kit, Hp Fast™, it is difficult to observe the gel color change upon a negative result at pH 5.8 and a positive result at pH 6.5. Therefore, the RUT results should be analyzed together with the performance of SYBR Green real-time PCR to confirm either a positive or a negative result.

The developed RUT was used to test gastric biopsy samples from eight canines, reading the results within 24 h, and compared with the commercial kit Hp Fast™. Of the eight canines assayed, one case tested positive by the developed RUT, commercial kit Hp Fast™ (GI supply, USA), and SYBR Green real-time PCR, while the remaining seven cases were negative. At 30 min, the developed test detected the target bacterium in the body, fundus, and antrum of the stomach based on the color change. This study was consistent with the previous studies by Sousa *et al*. [15] that used a RUT to detect *H. pylori* in the stomach antrum, body, and fundus of 32 cats from gastric biopsies. The detection of the RUT in cats can also be detected at 30 min. In this study, the histopathological diagnosis in the positive case correlated with symptoms of chronic enteritis duodenitis while the other seven canines gave negative results but had symptoms of ulcerative eosinophilic gastritis (Table-2). When confirmed by SYBR Green real-time PCR, it was found that *H. pylori* tested negative at 9.3×10^1^ copies and a Cq value at 39.58. It was previously reported by Bermejo *et al*. [23], with human patients with gastroenteritis, that positive RUT results required *H. pylori* infection at a minimum of 10,000 CFU/mL. Positive results in RUT assays are strongly dependent on the number of *H. pylori* in a specimen.

The specificity of the developed RUT assays was tested using a bacterial concentration of 150×10^6^ cells/mL (McFarland Standard 0.5); the results showed positivity for *H. pylori* ATCC 43504 (Figure-5a). The time at which the test turns positive depends on the concentration of bacteria and the urease [22,24]. The urease-positive bacteria used in prior RUT-specificity research included *S. pseudintermedius*, *Proteus* spp., *P. aeruginosa*, and *K. pneumoniae* [25]. At the same time, groups of bacteria capable of producing urease were negative (Figure-5b-e). The previous reports showed that the developed RUT could detect a minimum of 0.1 mg/mL urease, so negative results may be due to the urease not being sufficiently released or because the concentration was <0.1 mg/mL. The urease-negative group included *Enterococcus* spp., *E. coli*, and *Salmonella* spp. (Figure-5f-h). Therefore, the developed RUT did not detect any other infection. However, it is specific to members of the genus *Helicobacter* that can produce urease and release the enzyme at a concentration >0.1 mg/mL.

The developed test kit can be stored at 4°C for 6 months with the lid tightly closed (Figure-6b). However, when stored at 25°C, the developed RUT dried out after 2 weeks and could not be used. Since the test kit has a gel-like state, it can evaporate and dry at elevated temperatures (Figure-6a). This study showed that methyl paraben interfered with the function of the developed RUT (Figure-6).

The developed RUT should be read within 24 h with a closed lid to avoid false-positive results from contamination by bacteria that are not *H. pylori*, such as *Proteus*, *Yersinia*, *Klebsiella*, and *Pseudomonas*, which can produce urease; urea is hydrolyzed to produce ammonia, leading to an increase in pH causing a positive test result [26]. In this study, flaws were found in the substance used as it was not only opaque white plastic, but also too thick, making it difficult to observe any color change. The observed color change was less clear than with the Hp Fast™ commercial kit. Furthermore, observation of the experiments required repeated opening and closing of lids, which was inconvenient and created a risk of microbial contamination. Therefore, it is recommended that clear and translucent plastic be used for convenience and to minimize contamination. We will continue to develop the test and find suitable materials.

## Conclusion

The developed RUT approach used the RUT technique to detect urease produced by *H. pylori*. The developed assay was sensitive to urease at a concentration of 0.1 mg/mL at 30 min. The results were analyzed with SYBR Green real-time PCR. The developed RUT could detect *H. pylori* ATCC 43504 at a minimum of 10² copies. The developed RUT should be read within 24 h, not only with closed lids but also upon storage at 4°C to avoid contamination from bacteria in the environment.

## Authors’ Contributions

JR: Principle investigator, designed the study, drafted, critically revised the manuscript, and collected samples. CH: Designed and managed this research, did laboratory works, and wrote the manuscript. KC: Participated in the design of the study and interpretation of the data. CP: Participated in the bacterial strains and culture conditions. All authors read and approved the final manuscript.
